# Respiratory Infections in Adults with Atopic Disease and IgE Antibodies to Common Aeroallergens

**DOI:** 10.1371/journal.pone.0068582

**Published:** 2013-07-19

**Authors:** Aino Rantala, Jouni J. K. Jaakkola, Maritta S. Jaakkola

**Affiliations:** 1 Center for Environmental and Respiratory Health Research, University of Oulu, Oulu, Finland; 2 Public Health, Institute of Health Sciences, University of Oulu, Oulu, Finland; 3 Respiratory Medicine Unit, Department of Medicine, Oulu University Hospital, Oulu, Finland; 4 Respiratory Medicine Unit, Institute of Clinical Medicine, University of Oulu, Oulu, Finland; Centre de Recherche Public de la Santé (CRP-Santé), Luxembourg

## Abstract

**Background:**

Atopic diseases, including allergic rhinitis, allergic dermatitis and asthma, are common diseases with a prevalence of 30–40% worldwide and are thus of great global public health importance. Allergic inflammation may influence the immunity against infections, so atopic individuals could be susceptible to respiratory infections. No previous population-based study has addressed the relation between atopy and respiratory infections in adulthood. We assessed the relation between atopic disease, specific IgE antibodies and the occurrence of upper and lower respiratory infections in the past 12 months among working-aged adults.

**Methods and Findings:**

A population-based cross-sectional study of 1008 atopic and non-atopic adults 21–63 years old was conducted. Information on atopic diseases, allergy tests and respiratory infections was collected by a questionnaire. Specific IgE antibodies to common aeroallergens were measured in serum. Adults with atopic disease had a significantly increased risk of lower respiratory tract infections (LRTI; including acute bronchitis and pneumonia) with an adjusted risk ratio (RR) 2.24 (95% confidence interval [CI] 1.43, 3.52) and upper respiratory tract infections (URTI; including common cold, sinusitis, tonsillitis, and otitis media) with an adjusted RR 1.55 (1.14, 2.10). The risk of LRTIs increased with increasing level of specific IgE (linear trend *P* = 0.059).

**Conclusions:**

This study provides new evidence that working-aged adults with atopic disease experience significantly more LRTIs and URTIs than non-atopics. The occurrence of respiratory infections increased with increasing levels of specific IgE antibodies to common aeroallergens, showing a dose-response pattern with LRTIs. From the clinical point of view it is important to recognize that those with atopies are a risk group for respiratory infections, including more severe LRTIs.

## Introduction

Atopic diseases, including allergic rhinitis, allergic dermatitis and asthma, are common diseases with a prevalence of 30–40% worldwide and are thus of great global public health importance [1]. Atopic response is characterized by lymphocyte T-helper type 2 (Th2)-dependent immunological inflammation where Th2-derived cytokines, such as interleukin (IL) 4 and IL-13, induce allergic inflammation and the production of allergen-specific immunoglobulin E (IgE) antibodies [2]. As a consequence of Th2 polarization, Th1 response that plays an important role in protection against infectious agents mainly through Interferon gamma has been claimed to diminish in allergic individuals, leading to impairment of host defense responses against microbial infections [3]–[5]. Based on these mechanisms atopic subjects may have a higher susceptibility to respiratory infections than non-atopic subjects.

Given the high prevalence of atopic individuals even a small increase in the occurrence of respiratory infections would imply a substantial impact on disease burden at population level. Previous epidemiologic studies investigating the occurrence of respiratory infections in atopic and non-atopic subjects have been conducted among children and young adults [6]–[8]. Based on our systematic literature search (see Methods) there are no previous population-based studies addressing potential susceptibility of atopic subjects to respiratory infections in adulthood. To fill in this gap in knowledge, we conducted a large population-based cross-sectional study of working-aged adults to assess the relations between atopic diseases and specific IgE antibodies and occurrence of respiratory infections in the past 12 months period.

## Methods

### Study design

We applied a population-based cross-sectional study design. A random sample of adults 21 to 63 years of age living in Pirkanmaa were drawn using the national population registry, which has full coverage of the population. The Pirkanmaa Hospital District is a geographically defined administrative area in South Finland with a population of 440 913 in 1997 when this study started. A total of 1016 subjects (response rate 80%) answered the questionnaire. After excluding six persons older than 64 years and two with incomplete questionnaire, our study population included 1008 subjects. Of these, 73.2% gave a serum sample. This population was collected to form the control population of the Finnish Environment and Asthma Study (FEAS), a population-based incident case-control study described previously [9]–[16]. All study subjects signed an informed consent form. The study was approved by the ethics committees of the Finnish Institute of Occupational Health and the Tampere University Hospital.

### Systematic literature search

We performed a systematic literature search of Ovid Medline database with free text (“Atopy” OR “Allergic disease” OR “Allergic patient” OR “Allergic” OR “Immunoglobulin E”) AND with Mesh term “Respiratory tract infections”. We limited the search for English language and humans studies. The search produced altogether 262 articles from which three studies [6]–[8] fulfilled the following criteria: it (i) was a longitudinal/cohort study or case-control study and (ii) reported on relations between atopic diseases and/or specific IgE antibodies and occurrence of respiratory infections. We further searched the reference lists of recent review articles as well as relevant articles identified in our search.

### Questionnaire

The study subjects filled in a self-administered questionnaire [9]–[17] that included six sections: 1) personal characteristics, 2) health information, including occurrence of respiratory infections in the past 12 months and details of atopic and respiratory diseases, 3) active smoking and secondhand tobacco smoke (SHS) exposure, 4) occupation and work environment, 5) home environment, and 6) dietary questions. The parts of the questionnaire that were used in this study are included online as Supporting Information (Questionnaire S1).

### Assessment of atopic disease and specific allergies

The determinants of interest were atopic disease and specific IgE antibodies to common aeroallergens or a combination of these. These were studied as indicators of atopic propensity. Atopic disease was defined as reported doctor-diagnosed allergic rhinitis and/or dermatitis and/or asthma. In addition, serum concentrations of allergen-specific IgE antibodies were used as indicators of atopy.

The questionnaire also inquired the following questions: *Have you ever undergone allergy testing, such as skin prick tests or allergy-antibody tests?* and *Did these tests reveal any allergies (i.e. were the results positive)?* Reported positive findings of allergy tests were used as an additional indicator of atopic tendency in combination with diagnosed allergic diseases. We analyzed association between Phadiatop score and reported positive allergy test and they were highly correlated (Cochran-Armitage Trend Test: P<0.001).

Participants gave serum samples at the laboratories of the Tampere University Hospital Laboratory Center. Serum was separated by centrifugation and stored at −20°C before shipping to Finnish Institute of Occupational Health for IgE antibody analyses. Specific IgE antibodies to common aeroallergens, including birch, timothy, grass, mugwort, cat, dog, horse, *Dermatophagoides pteronyssinus*, and *Aspergillus fumigatus* were analyzed from the serum samples by the immunoassay system Pharmacia UniCAP (Pharmacia Diagnostics Ab, Uppsala, Sweden; www.unicapinvitrosight.com) [18] giving Phadiatop scores [13]. Phadiatop was expressed as a score from 0–6 as graded by the UniCAP Phadiatop system, with the limits as follows: score 0 = 0–0.34 kU/L, score 1 = 0.35–0.69 kU/L, score 2 = 0.70–3.49 kU/L, score 3 = 3.50–17.4 kU/L, score 4 = 17.5–49.9 kU/L, score 5  = 50–100 kU/L, and score 6 = greater than 100 kU/L.

### Assessment of the occurrence of respiratory infections

The main outcomes were occurrences of lower and upper respiratory infections assessed based on the following question: *How often did you experience the following infections during the past 12* *months?* The list of infections included common cold, tonsillitis, sinusitis, otitis media, acute bronchitis and pneumonia. The infections were dichotomized according to at least one infection (≥1) versus no infection, except that common cold was categorized as at least two infections (≥2) versus 0–1 infection. Respiratory infections were classified as lower respiratory tract infections (LRTI) including acute bronchitis and pneumonia, and upper respiratory tract infections (URTI) including common cold, tonsillitis, sinusitis and otitis media.

### Statistical methods

We studied the relations between atopic disease *per se* as well as in combination with measured specific IgE antibodies and occurrence of respiratory tract infections by estimating risk ratios (RR) with 95% confidence intervals (CI) from generalized linear model applying PROC GENMOD (SAS v9.3, SAS Institute Inc., Cary, NC, USA) using Poisson regression. The occurrence of infections was compared between subjects with one or several atopic diseases and subjects without any of the atopic diseases (the reference category). Specific IgE was expressed as a Phadiatop score reflecting the IgE antibody levels to common aeroallergens, and the risk of infections among those with higher scores was compared with the score 0 (the reference category). Sex, age, education (as an indicator of socio-economic status), personal smoking, and exposure to SHS in the home or at work were adjusted for as covariates. We tested the adjusted linear trend of the risk of URTIs and LRTIs according to the levels of specific IgE based on binomial proportion and their standard errors derived from Poisson regression (proc genmod indentity link). In addition, we performed the main analyses stratified by gender which we present in Tables S1, S2 and S3. However, these should be interpreted with caution as this stratification reduces the numbers in specific cells.

## Results

### Characteristics of the study population

Table 1 presents characteristics of the total study population and stratified by gender. The prevalence of allergic rhinitis, dermatitis and asthma (previous or current) in this working-aged population was 21.6% (n = 218), 34.1% (n = 344) and 7.5% (n = 76), respectively.

**Table 1:** Characteristics of the total study population and stratified by gender, The Finnish Environment and Asthma Study (FEAS).

		Female	Male	All
Characteristic		N (%)	N (%)	N (%)
Total		540	468	1008
Age (years)	21–29	89 (16.5)	59 (12.6)	148 (14.7)
	30–39	133 (24.6)	105 (22.4)	238 (23.6)
	40–49	150 (27.8)	126 (26.9)	276 (27.4)
	50–59	126 (23.3)	138 (29.5)	264 (26.2)
	60–64	42 (7.8)	40 (8.6)	82 (8.1)
Education^a^	No vocational schooling	91 (17.0)	74 (15.9)	165 (16.5)
	Vocational course	64 (11.9)	51 (10.9)	115 (11.5)
	Vocational institution	134 (25.0)	154 (33.1)	288 (28.7)
	College-level education	167 (31.1)	118 (25.3)	285 (28.4)
	University or corresponding	81 (15.1)	69 (14.8)	150 (15.0)
Smoking^b^	No	340 (63.1)	182 (39.0)	522 (51.9)
	Ex	90 (16.7)	132 (28.3)	222 (22.1)
	Current	109 (20.2)	153 (32.8)	262 (26.0)
SHS in the workplace		37 (7.4)	103 (23.3)	140 (14.8)
SHS in the home		26 (4.8)	29 (6.3)	55 (5.5)

Abbreviations: N, number; SHS, secondhand tobacco smoke.

a Information on education was missing for 5 subjects.

b Information on smoking was missing for 2 subjects.

### Association between atopic disease and IgE antibodies

The proportion of subjects with atopic disease increased significantly with higher IgE levels (i.e. Phadiatop scores 1–6) (Cochran-Armitage Trend Test: *P*<0.001). The risk of atopic disease was observed to increase with Phadiatop score showing a dose-response pattern: adjusted RR 1.36 (95% CI: 1.01, 1.82) for score 1–2, 1.93 (1.44, 2.57) for score 3–4, and 1.91 (0.77, 4.73) for score >4 (Table 2). The RRs of atopic disease related to the different Phadiatop scores were slightly higher among men than among women (Table S1).

**Table 2:** Association between specific IgE antibody levels and atopic disease, The Finnish Environment and Asthma Study (FEAS).

Phadiatop score	Subjects with atopic disease	Subjects with no atopic disease		
	N (%)	N (%)	RR (95% CI)	RR^a^ (95% CI)
Total^b^	350	378		
0	227 (64.9)	315 (83.3)	1	1
1–2	58 (16.6)	46 (12.2)	1.33 (1.00, 1.78)	1.36 (1.01, 1.82)
3–4	60 (17.1)	16 (4.2)	1.89 (1.42, 2.51)	1.93 (1.44, 2.57)
>4	5 (1.4)*	1 (0.3)	1.99 (0.82, 4.83)	1.91 (0.77, 4.73)

Abbreviations: CI, confidence interval; IgE, immunoglobulin E; N, number; RR, risk ratio.

* Cochran-Armitage Trend Test, 2-sided *P* value <0.001.

a Risk ratios adjusted for sex, age, education, smoking and SHS exposure (work/home).

b Total number of atopic and non-atopic subjects having Phadiatop result.

### Atopic disease and the risk of respiratory infections

Results in Table 3 show that subjects with atopic disease experienced more commonly both LRTIs and URTIs in the past 12 months compared to subjects with no atopic disease (12.2% vs. 5.9% and 24.3% vs. 13.3%, respectively). Atopic disease was related to an increased risk of both LRTIs (adjusted RR  = 2.24, 95% CI: 1.43, 3.52) and URTIs (adjusted RR  = 1.55, 95% CI: 1.14, 2.10). Consistent with this, atopic disease was significantly related to increased risk of specific respiratory infections including acute bronchitis, common cold, sinusitis and otitis media. The RR related to atopic disease was also increased for pneumonia and tonsillitis, but these effects did not reach statistical significance, probably because of smaller occurrences of these infections. Gender-stratified results of the relations between atopic disease and respiratory infections are shown in Table S2. The RR of LRTIs was higher among men than among women.

**Table 3:** Risk of respiratory infections in the past 12 months in subjects with atopic disease and those with no atopic disease, The Finnish Environment and Asthma Study (FEAS).

Infection^a^	Subjects with atopic disease	Subjects with no atopic disease		
	N (%)	N (%)	RR (95% CI)	RR^b^ (95% CI)
Total^c^	441	526		
LRTIs	54 (12.2)	31 (5.9)	2.08 (1.34, 3.23)	2.24 (1.43, 3.52)
Acute bronchitis	54 (12.2)	30 (5.7)	2.15 (1.37, 3.35)	2.32 (1.47, 3.66)
Pneumonia	3 (0.7)	2 (0.4)	1.79 (0.30, 10.71)	2.10 (0.33, 13.18)
URTIs	107 (24.3)	70 (13.3)	1.82 (1.35, 2.46)	1.55 (1.14, 2.10)
Common cold	172 (39.0)	152 (28.9)	1.35 (1.09, 1.68)	1.30 (1.04, 1.62)
Tonsillitis	23 (5.2)	19 (3.6)	1.44 (0.79, 2.65)	1.23 (0.66, 2.28)
Sinusitis	77 (17.5)	47 (8.9)	1.95 (1.36, 2.81)	1.57 (1.08, 2.26)
Otitis media	24 (5.4)	12 (2.3)	2.39 (1.19, 4.77)	2.28 (1.12, 4.61)

Abbreviations: CI, confidence interval; LRTI, lower respiratory tract infections; N, number; RR, risk ratio; URTI, upper respiratory tract infections.

a ≥1 infection. Common cold ≥2 infections.

b RR adjusted for sex, age, education, smoking and SHS exposure (work/home).

c Total number of atopic and non-atopic subjects. Information on infections was missing for 41 subjects.

The risk of LRTIs was observed to be even higher in those subjects who reported atopic disease in combination with positive allergy tests with an adjusted RR 2.79 (95% CI: 1.66, 4.70). URTIs were also increased, although the 95% CI included unity (adjusted RR  = 1.42, 95% CI: 0.97, 2.07) (Table 4).

**Table 4:** Risk of lower respiratory tract infections and upper respiratory tract infections during the past 12 months among subjects having atopic disease and high specific IgE level or positive allergy tests, The Finnish Environment and Asthma Study (FEAS).

		Lower respiratory tract infections		Upper respiratory tract infections	
Atopic manifestation	N	RR (95% CI)	RR^a^ (95% CI)	RR (95% CI)	RR^a^ (95% CI)
Atopic disease with positive allergy tests	199	2.56 (1.55, 4.23)	2.79 (1.66, 4.70)	1.77 (1.23, 2.57)	1.42 (0.97, 2.07)
Atopic disease with high specific IgE level^b^	119	2.14 (1.15, 3.96)	2.23 (1.19, 4.18)	1.52 (0.95, 2.41)	1.28 (0.80, 2.05)

Abbreviations: CI, confidence interval; N, number; RR, risk ratio.

a Risk ratios adjusted for sex, age, education, smoking and SHS exposure (work/home).

^b^ High specific IgE level  =  Phadiatop score 1–6.

In order to evaluate the temporal relation between the occurrence of atopic disease and respiratory infections we estimated the relations between atopic disease and occurrence of infections by stratifying the analysis by the time of diagnosis of atopic disease (over 12 months ago and in past 12 months). The adjusted RR of LRTIs was 1.99 (95% CI: 1.20, 3.28) in subjects whose atopic disease started over 12 months ago and 2.20 (1.29, 3.75) in subjects whose atopic disease was diagnosed in the past 12 months. The corresponding adjusted RRs of URTIs were 1.73 (1.22, 2.45) and 1.95 (1.35, 2.82), respectively.

We also analyzed the risk of infections according to the different atopic diseases (Table 5). Asthma and dermatitis significantly increased the risk of LRTIs (adjusted RR  = 4.69, 95% CI: 2.64, 8.35 and RR  = 1.82, 95% CI: 1.08, 3.07, respectively) and dermatitis significantly increased also the risk of URTIs (adjusted RR  = 1.62, 95% CI: 1.16, 2.26). In addition, the RR of both LRTIs and URTIs related to rhinitis were increased, but these did not reach statistical significance.

**Table 5:** Risk of respiratory infections in the past 12 months according to different atopic diseases, The Finnish Environment and Asthma Study (FEAS).

		Lower respiratory tract infections		Upper respiratory tract infections	
	N	RR (95% CI)	RR^a^ (95% CI)	RR (95% CI)	RR^a^ (95% CI)
Total^b^	967				
Non-atopic	526	1	1	1	1
Asthma	73	4.66 (2.65, 8.20)	4.69 (2.64, 8.35)	1.85 (1.10, 3.11)	1.62 (0.96, 2.74)
Rhinitis	210	1.12 (0.43, 2.88)	1.24 (0.48, 3.23)	1.38 (0.78, 2.46)	1.21 (0.68, 2.17)
Dermatitis	337	1.69 (1.01, 2.81)	1.82 (1.08, 3.07)	1.93 (1.39, 2.67)	1.62 (1.16, 2.26)

Abbreviations: CI, confidence interval; N, number; RR, risk ratio.

a Risk ratios adjusted for sex, age, education, smoking and SHS exposure (work/home).

b Information on infections was missing for 41 subjects.

### Specific IgE antibodies and the risk of respiratory infections

The risk of LRTIs increased with increasing specific IgE measured as Phadiatop score (adjusted linear trend *P* = 0.059) suggesting a dose response pattern with specific IgE. The risk of URTIs was also the highest among those with Phadiatop score of more than 4, although the 95% CIs of the adjusted RRs included unity and the trend was not linear (adjusted linear trend *P* = 0.555) (Table 6). The RR of LRTIs seemed to be higher among men than women with the Phadiatop score 3–4, although the numbers in specific cells were small (Table S3).

**Table 6:** Risk of lower and upper respiratory tract infections (≥1 infection) in the past 12 months according to the levels of specific IgE antibodies, The Finnish Environment and Asthma Study (FEAS).

Specific IgE	N	Subjects with infections, n (%)	RR (95% CI)	RR^a^ (95% CI)
Total	700			
Lower respiratory tract infections				
0	519	40 (7.7)	1	1
1–2	103	10 (9.7)	1.26 (0.63, 2.52)	1.26 (0.63, 2.54)
3–4	73	10 (13.7)	1.78 (0.89, 3.55)	1.80 (0.89, 3.65)
>4	5	1 (20.0)*	2.60 (0.36, 18.88)	3.40 (0.44, 26.38)
Upper respiratory tract infections				
0	519	103 (19.9)	1	1
1–2	103	20 (19.4)	0.98 (0.61, 1.58)	1.04 (0.64, 1.68)
3–4	73	12 (16.4)	0.83 (0.46, 1.51)	0.85 (0.46, 1.56)
>4	5	2 (40.0)**	2.02 (0.50, 8.17)	1.87 (0.44, 7.85)

Abbreviations: CI, confidence interval; IgE, immunoglobulin E; n, number; RR, risk ratio.

a Risk ratios adjusted for sex, age, education, smoking and SHS exposure (work/home).

*P* value for the adjusted linear trend test: * 0.059, ** 0.555.

The risk of LTRIs significantly increased among subjects with atopic disease in combination with high specific IgE levels with an adjusted RR of 2.23 (95% CI: 1.19, 4.18). URTIs were also increased in such subjects with an adjusted RR of 1.28 (95% CI: 0.80, 2.05), although this did not reach statistical significance (Table 4).

## Discussion

### Main Findings

The results of our large population-based study show that working-aged adults with atopic disease experience consistently more of both LRTIs and URTIs compared to adults with no atopic disease. Subjects with asthma were at the highest risk of LRTIs, experiencing about four times more LRTIs than those without asthma. The risk of infections was even higher when the subject with atopic disease reported positive allergy tests. Furthermore, the risk of infections seemed to increase according to increasing levels of specific IgE, suggesting a dose-response pattern and supporting the hypothesis that allergies are linked to increased susceptibility to infections. The risk of LRTIs and URTIs was also related to atopic dermatitis and allergic rhinitis, although the 95% CI of the latter effect estimate included unity. It is known that atopic diseases share similar impaired immunological mechanisms that may increase susceptibility to respiratory infections [5], [19]. It is possible that dermatitis is a more consistent manifestation of atopic constitution and therefore the association with respiratory infections could be stronger compared to rhinitis.

Recently, we published from the Finnish Environment and Asthma Study including the incident (new) cases that respiratory infections were a strong determinant for adult-onset asthma [20]. The strongest risk was seen in those who had atopic disease and had experienced LRTIs during the last 12 months. Taking together the findings of these two studies, it seems that persons with allergy are more prone to respiratory infections and more susceptible to the effects of LRTIs on the risk of asthma.

The mechanisms underlying the susceptibility to microbial infections observed in this study among atopic subjects are poorly understood. Allergic patients typically present a Th2 polarization and hence it is suggested that the protective Th1 response is reduced. In addition, Th2-driven responses are believed to impair the innate immunity mediated host defense responses against microbial infections [5]. Th2-mediated cytokines, such as IL-4 and IL-13, induce airway inflammation that facilitates microbial attachment to the airway epithelia. In addition, IgE antibodies have been shown to suppress neutrophil adhesion that is an important feature of innate immune responses [21]. The present results show that atopic disease and increased levels of specific IgE are significantly related to the risk of both LRTIs and URTIs. We found that those atopic persons who have high IgE level could have stronger inflammation e.g. in airways leading to even stronger susceptible to infections.

We found that the association between atopy and lower respiratory infections seemed to be stronger among men than women. However, the present sex stratified results should be interpreted with caution as the numbers in specific cells are small due to the stratification.

### Validity of results

The study population was randomly selected from a defined geographic area and the response rate was high (80%). Thus, our results are well generalizable and any major selection bias is unlikely.

Information on the atopic disease was based on reporting allergic rhinitis, allergic dermatitis or asthma diagnosed by a doctor in the questionnaire. Self-reported atopic diseases could include some misclassification, especially if the access to health care system was compromised and related to environmental and dietary conditions that determine their occurrence. The Finnish health care system has a high coverage for the entire population and the majority of the individuals with atopic diseases are likely to receive medical attention. Diagnosis of asthma is further enhanced by reimbursement of medication by the National Social Insurance Institution, which is a strong incentive for an appropriate clinical diagnosis. In addition, we used measurements of specific IgE in combination with reported diseases to strengthen the definition of atopic disease and the risk of both URTIs and LRTIs were increased in this group. Specific IgE antibodies were measured from serum samples using a validated commercial UniCAP system [18]. This method is easier to conduct in a standardized way than skin testing. Phadiatop includes in Finland the following aeroallergens: birch, timothy grass, mugwort, cat, dog, horse, *Dermatophagoides pteronyssinus*, and *Aspergillus fumigatus*. The selection of this panel reflects well the most common or otherwise important aeroallergies in Finland. The sensitivity of Phadiatop has been between 0.93 and 0.99 and the specificity between 0.87 and 0.94 by using a clinical diagnosis of atopy as the gold standard [13], [18]. In our own study, we found a dose-response pattern in increasing risk of atopic disease with increasing level of specific IgE.

Information about the frequency and type of respiratory infections during the past 12 months was assessed based on reporting in the questionnaire. Although there may be some misclassification, in our opinion it is likely to be non-differential or atopic subjects tend to assume infections as exacerbations of their allergic symptoms and both of these would lead to underestimation of the real effect. We did not have data on specific infectious agents, but this information is not so relevant from the public health preventive point of view. We collected data concerning different types of infections, including common cold, tonsillitis, sinusitis, otitis media, acute bronchitis or pneumonia, but our main analyses concentrated on LTRIs and URTIs, as it seems plausible that people are able to recall well if they have had a severe respiratory infection, such as acute bronchitis or pneumonia, or a milder upper respiratory infection, although the exact diagnosis may be more difficult to remember.

In the multivariate analyses, all the relations were adjusted for a number of potential confounders to eliminate these factors as explanations for our results.

### Synthesis with previous knowledge

In our systematic literature search, no previous population-based study addressing the relations between atopic disease and respiratory infections covering the working aged adults could be identified. Only a few studies had addressed this question in children or young adults. Cirillo and co-workers [7] found in Italian Navy soldiers (with the mean age of 22.7 years) that those with allergic rhinitis had a higher rate of infections. The risk of severe respiratory infections (among which they included middle ear infections, paranasal sinusitis and lower airway infections) was higher than the risk of mild infections. Their results are thus consistent with ours covering adult age. We found that allergic rhinitis was a weaker risk factor for infections than asthma or allergic dermatitis. The Italian study did not address these other forms of allergic diseases. Another Italian study of 3–6 years old children referred to an outpatient clinic reported a higher occurrence of respiratory infections among allergic compared to non-allergic children [6]. Kvaerner and co-workers [8] studied the occurrence of URTIs in a population-based cross-sectional study of Norwegian children aged 4 to 5 years and found that infections were associated with atopic disease. A British study followed for 3–4 months 76 cohabiting couples, one having atopic asthma and the other being healthy, and took repeatedly nasal samples for rhinovirus analysis [22]. They did not find differences in rhinovirus-related URTIs, but asthmatics were more likely to have LRTIs. In addition to studies focusing on respiratory tract infections, a population-based case-control study using registries as sources of information found increased risk of serious pneumococcal disease (including sepsis, meningitis and pneumonia) in both children and adults with atopic disease other than asthma [23]. Association between levels of specific IgE antibodies and respiratory infections has not been studied previously.

### Conclusions

We present here new evidence that working-aged adults with atopic disease are more susceptible to both LRTIs and URTIs than adults with no atopic disease. In addition, the occurrence of respiratory infections increased with increasing levels of specific IgE antibodies to common aeroallergens, showing a dose-response pattern with LRTIs. From the preventive and clinical point of view it is important to recognize that those with atopies are a risk group for respiratory infections, including more severe lower respiratory infections. Relevant questions for the future are to study whether effective treatment of allergic diseases could prevent respiratory infections, and to consider if all those with atopic diseases should be subject to preventive measures, such as vaccinations.

## Supporting Information

Questionnaire S1 **The parts of the questionnaire that were used in this study, The Finnish Environment and Asthma Study (FEAS).**
(DOC)

Table S1 **Association between specific IgE antibody levels and atopic disease stratified by gender, The Finnish Environment and Asthma Study (FEAS).**
(DOCX)

Table S2 **Risk of respiratory infections in the past 12** **months in subjects with atopic disease and those with no atopic disease stratified by gender, The Finnish Environment and Asthma Study (FEAS).**
(DOCX)

Table S3 **Risk of lower and upper respiratory tract infections (≥1 infection) in the past 12** **months according to the levels of specific IgE antibodies stratified by gender, The Finnish Environment and Asthma Study (FEAS).**
(DOCX)
