# Supplementary material for: Respiratory Infections in Adults with Atopic Disease and IgE Antibodies to Common Aeroallergens
Source: PLoS One. 2013 Jul 19;8(7):e68582. doi: 10.1371/journal.pone.0068582 (PMC3716702; doi:10.1371/journal.pone.0068582)
Supplement: Questionnaire S1 — The parts of the questionnaire that were used in this study, The Finnish Environment and Asthma Study (FEAS). (DOC) [file pone.0068582.s001.doc]

**Center for Environmental and Respiratory Health Research**

**The Finnish Institute of Occupational Health**

### The University of Helsinki, Department of Public Health

### FINNISH ENVIRONMENT AND ASTHMA STUDY

The study evaluates the role of factors in the work and home environments in the development of asthma, and their other potential effects on the respiratory health. It is important that everybody who receives this questionnaire participates to ensure the validity of the research results! All the answers will be treated with absolute confidentiality, and the report will consider the results only by groups. To ensure the success of the study, it is important that you answer all the questions to the best of your ability. Circle the number corresponding to the most appropriate alternative or write your answer on the line next to or under the question. If a question seems to be unclear, give your feedback at the end of the questionnaire, but don’t leave the question unanswered.

### PERSONAL DATA

Questionnaire filled out on ____, ____, 199__, at ____ o’clock

(day, month)

Study identification no. ______(stick-on label, or researcher fills out)

Name:

Address:

Telephone:

1. Date of birth: ____, ____, 199__

(day, month)

1. Age: ___ years
2. Gender: 1 man 2 woman
3. Education:
4. no vocational schooling
5. vocational course
6. vocational institute
7. college-level education
8. university or corresponding
9. other, what: ____

### DATA ON HEALTH STATUS

## **INFECTIONS**

1. How often did you have the following infections during the past year (12 months) and the past 3 months? (Mark 0, if not at all)

Number of times

During the past 12 months During the past 3 months

1. Common cold (’flu’) ________ _________
2. Tonsillitis
3. Sinus infection
4. Infection of the middle ear
5. Bronchitis
6. Pneumonia
7. Diarrhea

#### ALLERGIES AND DOCTOR-DIAGNOSED DISEASES

# 17. Have you ever had allergic rhinitis (for example hay fever) diagnosed by a doctor

1. yes, during the past 12 months
2. yes, over 12 months ago only
3. no, never

18. Have you ever had allergic dermatitis (skin problems) diagnosed by a doctor?

1. yes, during the past 12 months
2. yes, over 12 months ago only
3. no, never

19. Have you ever had asthma diagnosed by a doctor?

1. yes, asthma diagnosed during the past 12 months
2. yes, asthma diagnosed over 12 months ago
3. no, never

28. Have you ever undergone allergy tests, for example, skin prick tests or allergy-antibody tests?

1 yes 2 no

If you answered yes, answer also questions 29 and 30, otherwise go straight to 31 (on page ).

29. Did these tests reveal any allergies (were the results positive)?

1 yes 2 no

### SMOKING AND ENVIRONMENTAL TOBACCO SMOKE

35. Do you smoke nowadays?

1. Yes, regularly (at least 1 cigarette a day or 25 g of pipe tobacco a month)
2. Yes, occasionally
3. No, I quit less than 12 months ago;

how many months ago? ____ months

1. No, I quit over a year ago;

how many years ago? ___ years

1. I have never smoked regularly

39. Did your coworkers smoke in your work site (in your own workspace) during the past year (12 months)?

1 no 2 yes

40. Has anyone smoked regularly in your home (indoors) during the past year (12 months) (excluding your own smoking)?

1. no
2. yes, on average

___ cigarettes a day

___ cigars a day

___ pipefuls a day

Please, check that you have answered all questions!

THANK YOU FOR TAKING THE TROUBLE TO ANSWER THIS QUESTIONNAIRE!

Please feel free to give your comments regarding this questionnaire on the lines below

______________________________________________________

______________________________________________________

© M.S. Jaakkola & J.J.K. Jaakkola
