# Supplementary material for: Respiratory Infections in Adults with Atopic Disease and IgE Antibodies to Common Aeroallergens
Source: PLoS One. 2013 Jul 19;8(7):e68582. doi: 10.1371/journal.pone.0068582 (PMC3716702; doi:10.1371/journal.pone.0068582)
Supplement: Table S1 — Association between specific IgE antibody levels and atopic disease stratified by gender, The Finnish Environment and Asthma Study (FEAS). (DOCX) [file pone.0068582.s002.docx]

**Table S1.** Association between specific IgE antibody levels and atopic disease stratified by gender, The Finnish Environment and Asthma Study (FEAS)

|  | **All** | | | **Female** | | | **Male** | | |
| --- | --- | --- | --- | --- | --- | --- | --- | --- | --- |
| **Specific IgE** | **N** | **RR (95% CI)** | **RR^a^ (95% CI)** | **N** | **RR (95% CI)** | **RR^b^ (95% CI)** | **N** | **RR (95% CI)** | **RR^b^ (95% CI)** |
| Total | 728 |  |  | 404 |  |  | 324 |  |  |
| 0 | 542 | 1 | 1 | 308 | 1 | 1 | 234 | 1 | 1 |
| 1-2 | 104 | 1.33 (1.00-1.78) | 1.36 (1.01-1.82) | 53 | 1.19 (0.81-1.74) | 1.17 (0.79-1.72) | 51 | 1.65 (1.06-2.56) | 1.67 (1.07-2.61) |
| 3-4 | 76 | 1.89 (1.42-2.51) | 1.93 (1.44-2.57) | 39 | 1.82 (1.26-2.63) | 1.81 (1.24-2.64) | 37 | 2.11 (1.34-3.31) | 2.16 (1.36-3.41) |
| >4 | 6 | 1.99 (0.82-4.83) | 1.91 (0.77-4.73) | 4 | 1.52 (0.48- 4.76) | 1.47 (0.46-4.72) | 2 | 3.12 (0.77-12.71) | 3.29 (0.77-14.14) |

Abbreviations: CI, confidence interval; IgE, immunoglobulin E; N, number; RR, risk ratio

^a^ Risk ratios adjusted for sex, age, education, smoking and SHS exposure (work/home)

^b^ Risk ratios adjusted for age, education, smoking and SHS exposure (work/home)
