# Supplementary material for: Respiratory Infections in Adults with Atopic Disease and IgE Antibodies to Common Aeroallergens
Source: PLoS One. 2013 Jul 19;8(7):e68582. doi: 10.1371/journal.pone.0068582 (PMC3716702; doi:10.1371/journal.pone.0068582)
Supplement: Table S2 — Risk of respiratory infections in the past 12 months in subjects with atopic disease and those with no atopic disease stratified by gender, The Finnish Environment and Asthma Study (FEAS). (DOCX) [file pone.0068582.s003.docx]

**Table S2.** Risk of respiratory infections in the past 12 months in subjects with atopic disease and those with no atopic disease stratified by gender, The Finnish Environment and Asthma Study (FEAS)

|  | **All (n=967)^a^** | | **Female (n=524)** | | **Male (n=443)** | |
| --- | --- | --- | --- | --- | --- | --- |
| **Infection^b^** | **RR (95% CI)** | **RR^c^ (95% CI)** | **RR (95% CI)** | **RR^d^ (95% CI)** | **RR (95% CI)** | **RR^d^ (95% CI)** |
| **LRTIs** | 2.08 (1.34-3.23) | 2.24 (1.43-3.52) | 1.75 (0.93-3.31) | 1.81 (0.95-3.45) | 2.68 (1.45- 4.96) | 2.92 (1.56- 5.44) |
| Acute bronchitis | 2.15 (1.37-3.35) | 2.32 (1.47-3.66) | 1.89 (0.98-3.63) | 1.97 (1.02-3.81) | 2.68 (1.45-4.96) | 2.92 (1.56-5.44) |
| Pneumonia | 1.79 (0.30-10.71) | 2.10 (0.33-13.18) | 0.85 (0.05-13.51) | 0.75 (0.04-13.29) | 3.64 (0.33-40.18) | - |
| **URTIs** | 1.82 (1.35-2.46) | 1.55 (1.14-2.10) | 1.53 (1.08-2.18) | 1.48 (1.04-2.12) | 1.66 (0.90-3.03) | 1.70 (0.92-3.13) |
| Common cold | 1.35 (1.09-1.68) | 1.30 (1.04-1.62) | 1.38 (1.03-1.85) | 1.36 (1.01-1.82) | 1.19 (0.84-1.70) | 1.22 (0.86-1.74) |
| Tonsillitis | 1.44 (0.79-2.65) | 1.23 (0.66-2.28) | 1.54 (0.74-3.21) | 1.46 (0.70-3.07) | 0.68 (0.18-2.57) | 0.71 (0.18-2.71) |
| Sinusitis | 1.95 (1.36-2.81) | 1.57 (1.08-2.26) | 1.55 (1.03-2.33) | 1.50 (0.99-2.25) | 1.82 (0.79-4.20) | 1.85 (0.80- 4.32) |
| Otitis media | 2.39 (1.19-4.77) | 2.28 (1.12-4.61) | 1.41 (0.62-3.22) | 1.39 (0.60-3.20) | 5.46 (1.48- 20.19) | 5.77 (1.54-21.61) |

Abbreviations: CI, confidence interval; LRTI, lower respiratory tract infections; n, number; RR, risk ratio; URTI, upper respiratory tract infections

^a^ Total number of atopic and non-atopic subjects. Information on infections was missing for 41 subjects.

^b^ ≥ 1 infection. Common cold ≥ 2 infections.

^c^ RR adjusted for sex, age, education, smoking and SHS exposure (work/home),

^d^ Risk ratios adjusted for age, education, smoking and SHS exposure (work/home)
