# Supplementary material for: Respiratory Infections in Adults with Atopic Disease and IgE Antibodies to Common Aeroallergens
Source: PLoS One. 2013 Jul 19;8(7):e68582. doi: 10.1371/journal.pone.0068582 (PMC3716702; doi:10.1371/journal.pone.0068582)
Supplement: Table S3 — Risk of lower and upper respiratory tract infections (≥1 infection) in the past 12 months according to the levels of specific IgE antibodies stratified by gender, The Finnish Environment and Asthma Study (FEAS). (DOCX) [file pone.0068582.s004.docx]

**Table S3.** Risk of lower and upper respiratory tract infections (≥ 1 infection) in the past 12 months according to the levels of specific IgE antibodies stratified by gender, The Finnish Environment and Asthma Study (FEAS)

|  | **All** | | | **Female** | | | **Male** | | |
| --- | --- | --- | --- | --- | --- | --- | --- | --- | --- |
| **Specific IgE** | **N** | **RR (95% CI)** | **RR^a^ (95% CI)** | **N** | **RR (95% CI)** | **RR^b^ (95% CI)** | **N** | **RR (95% CI)** | **RR^b^ (95% CI)** |
| Total | 700 |  |  | 393 |  |  | 307 |  |  |
| **Lower respiratory tract infections** | | | | | | | | | |
| 0 | 519 | 1 | 1 | 298 | 1 | 1 | 221 | 1 | 1 |
| 1-2 | 103 | 1.26 (0.63-2.52) | 1.26 (0.63-2.54) | 53 | 1.02 (0.35-2.97) | 1.06 (0.36-3.11) | 50 | 1.47 (0.58-3.71) | 1.67 (0.65-4.29) |
| 3-4 | 73 | 1.78 (0.89-3.55) | 1.80 (0.89-3.65) | 38 | 0.71 (0.17-3.03) | 0.70 (0.16-3.05) | 35 | 2.81 (1.22-6.45) | 2.95 (1.26-6.89) |
| >4 | 5 | 2.60 (0.36-8.88) | 3.40 (0.44-26.38) | 4 | 3.39 (0.46-25.12) | 5.52 (0.65-47.29) | 1 | - | - |
| **Upper respiratory tract infections** | | | | | | | | | |
| 0 | 519 | 1 | 1 | 298 | 1 | 1 | 221 | 1 | 1 |
| 1-2 | 103 | 0.98 (0.61-1.58) | 1.04 (0.64-1.68) | 53 | 0.91 (0.50-1.62) | 0.92 (0.51-1.66) | 50 | 1.41 (0.60-3.29) | 1.49 (0.63-3.53) |
| 3-4 | 73 | 0.83 (0.46-1.51) | 0.85 (0.46-1.56) | 38 | 0.77 (0.37-1.60) | 0.75 (0.36-1.56) | 35 | 1.15 (0.40-3.33) | 1.17 (0.40-3.43) |
| >4 | 5 | 2.02 (0.50-8.17) | 1.87 (0.44-7.85) | 4 | 1.84 (0.45-7.48) | 2.01 (0.47-8.55) | 1 | - | - |

Abbreviations: CI, confidence interval; IgE, immunoglobulin E; N, number; RR, risk ratio

^a^ Risk ratios adjusted for sex, age, education, smoking and SHS exposure (work/home)

^b^ Risk ratios adjusted for age, education, smoking and SHS exposure (work/home)
